# Author’s response: False-positive results with rapid diagnostic test for dengue in Thailand

**DOI:** 10.2807/1560-7917.ES.2019.24.21.1900309

**Published:** 2019-05-23

**Authors:** Anu Kantele

**Affiliations:** 1 Professor of Infectious Diseases, Inflammatory Center, Helsinki University Hospital and Helsinki University

**Keywords:** chikungunya, CHIKV, dengue, DENV, zika, ZIKV, rapid diagnostic test, RDT, Thailand

I thank Javelle et al. for the methodological remarks [1] they made about my recent paper describing a cluster of chikungunya cases among visitors to Thailand [2]. Their letter concerns the positive dengue rapid diagnostic test (RDT) results recorded for two of our patients at the local hospital at their holiday destination. Unfortunately, no details are available on the RDT that was used, yet—as Javelle et al. point out—those commonly applied in dengue diagnostics lack sensitivity and specificity [3].

The dengue virus (DENV) IgG determination carried out at HUSLAB, Helsinki University Hospital, which is responsible for most of the dengue diagnostics in Finland, is based on an in-house immunofluorescence assay (IFA) that, together with a commercial IgM EIA testing, would have shown DENV antibody response at the time of sampling in Finland [4]. Moreover, the IgG test is strongly cross-reactive between flaviviruses. An early case of congenital Zika virus (ZIKV) infection, for example, has been identified using this DENV-IgG-IFA test [5]. Similar IgG titres are typically obtained for both the DENV and ZIKV antigens, regardless of the infecting virus [6]. Strong cross-reactivity is also seen with other mosquito-borne flaviviruses. For these reasons, I am confident that the negative DENV-IgG IFA results rule out a recent mosquito-borne flavivirus, particularly DENV or ZIKV infection, confirming that the patients did not have a DENV/ZIKV co-infection with CHIKV. Nevertheless, clinicians should be reminded about the possibility of co-infections with ZIKV, DENV and CHIKV, as they all cause broadly similar symptoms and circulate in the same areas [7,8].

## References

[1] Javelle E Gautret P Leparc-Goffart I Letter to the editor: False positive results with rapid diagnostic tests (RDT) for dengue. Euro Surveill. 2019; 24(21):1900304 Forthcoming. 10.2807/1560-7917.ES.2019.24.21.1900304 PMC6540641 31138364

[2] Kantele A Travellers as sentinels of chikungunya epidemics: a family cluster among Finnish travellers to Koh Lanta, Thailand, January 2019. Euro Surveill. 2019;24(11):1900162. 10.2807/1560-7917.ES.2019.24.11.1900162 30892179 PMC6425551

[3] Hunsperger EA Yoksan S Buchy P Nguyen VC Sekaran SD Enria DA Evaluation of commercially available diagnostic tests for the detection of dengue virus NS1 antigen and anti-dengue virus IgM antibody. PLoS Negl Trop Dis. 2014;8(10):e3171. 10.1371/journal.pntd.0003171 25330157 PMC4199549

[4] Erra EO Korhonen EM Voutilainen L Huhtamo E Vapalahti O Kantele A Dengue in travelers: kinetics of viremia and NS1 antigenemia and their associations with clinical parameters. PLoS One. 2013;8(6):e65900. 10.1371/journal.pone.0065900 23755291 PMC3670861

[5] Driggers RW Ho CY Korhonen EM Kuivanen S Jääskeläinen AJ Smura T Zika virus infection with prolonged maternal viremia and fetal brain abnormalities. N Engl J Med. 2016;374(22):2142-51. 10.1056/NEJMoa1601824 27028667

[6] Jääskeläinen AJ Korhonen EM Huhtamo E Lappalainen M Vapalahti O Kallio-Kokko H Validation of serological and molecular methods for diagnosis of zika virus infections. J Virol Methods. 2019;263:68-74. 10.1016/j.jviromet.2018.10.011 30342068

[7] Furuya-Kanamori L Liang S Milinovich G Soares Magalhaes RJ Clements AC Hu W Co-distribution and co-infection of chikungunya and dengue viruses. BMC Infect Dis. 2016;16(1):84. 10.1186/s12879-016-1417-2 26936191 PMC4776349

[8] Waggoner JJ Gresh L Vargas MJ Ballesteros G Tellez Y Soda KJ Viremia and clinical presentation in Nicaraguan patients infected with zika virus, chikungunya Virus, and dengue virus. Clin Infect Dis. 2016;63(12):1584-90. 10.1093/cid/ciw589 27578819 PMC5146717

